# Molecular Research on Coronavirus: Pathogenic Mechanisms, Antiviral Drugs, and New Vaccines

**DOI:** 10.3390/ijms25116172

**Published:** 2024-06-04

**Authors:** Mengjia Zhang, Yifei Lang, Wentao Li

**Affiliations:** 1 National Key Laboratory of Agricultural Microbiology, Hubei Hongshan Laboratory, College of Veterinary Medicine, Huazhong Agricultural University, Wuhan 430070, China; zhangmengjia0210@mail.hzau.edu.cn; 2 College of Veterinary Medicine, Sichuan Agricultural University, Chengdu 611130, China; y_langviro@163.com

Since the COVID-19 outbreak in 2019, five coronaviruses have been found to infect humans, including SARS-CoV (severe acute respiratory syndrome coronavirus) [1], HCoV-NL63 (HCoV-NL63) [2], HCoV-HKU1 (HCoV-HKU1) [3], MERS-CoV (Middle East respiratory syndrome coronavirus) [4], and SARS-CoV-2 (severe acute respiratory syndrome coronavirus 2) [5]. In addition to human CoVs, porcine CoVs such as TGEV (transmissible gastroenteritis virus) [6], PEDV (porcine epidemic diarrhea virus) [7], PDCoV (porcine deltacoronavirus) [8], PHEV (porcine hemagglutinating encephalomyelitis virus), and SADS-CoV (swine acute diarrhea syndrome coronavirus) [9] also cause severe disease in domestic animals. Consequently, coronaviruses pose significant threats to both public and animal health, as well as to economic stability. Over the past decades, scientists have made significant progress in exploring multiple aspects of coronaviruses and their control, including pathogenic mechanisms, antiviral drugs, and new vaccines [10,11,12]. Reflecting the rapid pace of technological development, this Special Issue, which contains four original articles and one review, highlights the latest advancements in these areas.

COVID-19 is recognized as a multisystem disease [13,14,15]. Vasculopathy generally, and endothelial dysfunction, hypercoagulability, and microthrombi in particular, can be induced by cytokine storms following infection and is a common factor in cardiovascular complications [16,17,18,19]. Recent research has shown that the risk of cardiovascular complications decreases following vaccination with BNT162b2, a safe and effective COVID-19 vaccine [20]. Wigner-Jeziorska et al. reported that convalescent individuals vaccinated with BNT162b2 showed an increased expression of MMP-7 (metalloproteinases 7), while non-vaccinated convalescent individuals showed an increased mRNA expression of ADAMTS1 (ADAM metallopeptidase with thrombospondin type 1 motif 1), elucidating the significant molecular impacts of SARS-CoV-2 infection and vaccination on angiogenesis [21]. Additionally, chronic COVID-19 patients suffer from various types of pain. Drawing from this, a review in this Special Issue analyzed existing microRNA data on chronic COVID-19 and proposed that microRNAs play a regulatory role in the IL-6/STAT3 proinflammatory axis and also make up the blood–nerve barrier, contributing to pain symptoms in chronic COVID-19. These findings suggest that microRNAs could be novel targets for COVID-19 treatment.

Recent research has identified an increasing number of animal coronaviruses capable of infecting humans or human cells, raising concerns about cross-species transmission. PDCoV has been detected in pediatric patients, leading to the onset of acute febrile illness [22]. The newly identified SADS-CoV, a type of animal coronavirus discovered in 2018, has been shown to efficiently replicate in various human cell lines [23,24]. Zhou and Zhang et al. successfully generated six monoclonal antibodies targeting the SADS-CoV S protein, among which three exhibited neutralizing activity and two showed hemagglutination inhibitory activity. These antibodies have potential as therapeutic agents against SADS-CoV infection, offering a novel approach for antiviral medication development for animal coronaviruses. Zeng and Wang focused on another clinically significant animal coronavirus, PEDV. Zeng et al. were the first to report that PEDV nsp14 negatively regulates GRP78, a marker protein of ER stress, revealing a novel pathogenic mechanism and identifying a promising target for anti-PEDV drug development. Wang et al. developed a bivalent genetically engineered vaccine providing protection against both Classical Swine Fever (CSF) and PED, two highly contagious viral diseases threatening the swine industry. They have developed a recombinant CSF vaccine C-strain that expresses the antigenic domains of PEDV, the S1N domain and the COE domain [25]. To enhance the efficacy of the vaccine, tissue plasminogen activator signal (tPAs) and the CARD domain of the signaling molecule VISA were introduced, resulting in the secretory expression of the S1NCOE protein and the increased induction of IFN-β in infected cells. The vaccine candidate significantly boosted PEDV-specific antibody production and upregulated PEDV-specific IFN-γ levels in vaccinated animals. Notably, vaccination with the engineered vaccine provided protection against virulent CSFV and PEDV challenges in pigs, highlighting its potential as a promising bivalent vaccine candidate that could optimize immunization protocols and improve disease prevention strategies in the swine industry.

We acknowledge all the authors’ valuable contributions to this Special Issue. The research findings and reviews compiled in this Special Issue provide updated insights into the pathogenic mechanisms of coronaviruses, and antiviral drugs and new vaccines for their treatment. The unique genomic structure and replication characteristics of coronaviruses make them more prone to evolution and recombination than other viruses, as evidenced by SARS-CoV-2. Therefore, continued investigation into the pathogenic mechanisms of coronaviruses and the development of antiviral strategies are imperative for a comprehensive understanding of this virus type and to effectively combat new viral threats.
